# P038. Effects of non-invasive vagus nerve stimulation on cerebral vasomotor reactivity in patients with chronic migraine during intercritical phase: a pilot study

**DOI:** 10.1186/1129-2377-16-S1-A62

**Published:** 2015-09-28

**Authors:** Riccardo Altavilla, Matteo Paolucci, Claudia Altamura, Fabrizio Vernieri

**Affiliations:** Headache Centre, Neurology Unit, University Campus Bio-Medico, Rome, Italy

## Introduction

Different strategies of neurostimulation have been developed as treatment tools for migraine. Among them, vagus nerve stimulation (VNS) can be performed both invasively and non-invasively. Recently, “Gammacore” has been approved as a non-pharmacological and non-invasive tool for headache, and a recent study demonstrated its efficacy in 22% of patients with acute migraine attacks[1].

Although the pathophysiology of migraine is not yet fully understood, many studies have shown a role of sterile inflammation of cerebral vessels and of the change in diameter of the intracranial arteries. Blood flow velocities and vasomotor reactivity (VMR) in patients suffering from migraine without aura in the intercritical phase were found either increased or normal compared to non-migraineurs healthy controls[2, 3]. Since the vagus nerve is the largest parasympathetic nerve of the body, it is probable that its neuromodulation can affect cerebral hemodynamics. The purpose of the study was to evaluate the effects of external vagus nerve stimulation on VMR of patients suffering from chronic migraine.

## Study design

We enrolled 20 patients aged between 18 and 65 years, suffering from chronic migraine and 20 healthy non-migraineur subjects matched for demographic characteristics. None of them assumed vaso-reactive drugs for at least 30 days before registration.

Subjects enrolled underwent registration of blood flow velocity in the middle cerebral artery bilaterally through transcranial Doppler (TCD), in the morning and fasting from food and caffeine. The monitoring was performed at baseline, during and after apnea lasting 30 seconds. After the recording, the subjects underwent external stimulation of the vagus nerve with “Gammacore” device for 90 seconds, and 20 minutes after we once again registered VMR through apnea test. VMR was calculated by means of Breath holding index according to the following formula[4]: VMR/apnea duration in seconds.

## Preliminary results

No statistically significant differences emerged comparing VMR before and after VNS in our population, irrespective of groups. No patient suffered from adverse event during or after VNS.

## Conclusions

Non-invasive VNS resulted safe and did not seem to influence VMR, neither in migraineurs nor in healthy volunteers. However, the small sample of our study population does not allow to draw definitive conclusions, hence the study will be further continued to extend sample size.

Written informed consent to publication was obtained from the patient(s).
